# Supplementary material for: Transcriptome Remodeling in Gradual Development of Inverse Resistance between Paclitaxel and Cisplatin in Ovarian Cancer Cells
Source: Int J Mol Sci. 2020 Dec 3;21(23):9218. doi: 10.3390/ijms21239218 (PMC7730278; doi:10.3390/ijms21239218)
Supplement: Supplementary file 1 [file ijms-21-09218-s001.zip › suppl/Supplementary materials_ijms970222 revised.pdf]

Table S1. Serial drug dilutions used in the cytotoxicity assay

Cell line	PTX serial dilution (nM)	CDDP serial dilution (μM)
A2780	1-1.5-2-3-5-7-10-25	1-1.5-2-3-5-7-10-25
A/4PTX-63d	5-7.5-10-12.5-15-25-50-100	0.25-0.5-0.75-1-2.5-5-7.5-10
A/8PTX-33d	5-7.5-10-12.5-15-25-50-100	0.25-0.5-0.75-1-2.5-5-7.5-10
A/16PTX-56d	25-50-75-100-150-175-200-300	0.25-0.5-0.75-1-2.5-5-7.5-10
A/32PTX-48d	10-50-150-175-200-300-500-600	0.02-0.1-0.2-0.4-0.6-0.8-1-4
A/64PTX-30d	10-50-150-175-200-250-300-400	0.02-0.1-0.2-0.4-0.6-0.8-1-4
A/128PTX-37d	50-100-150-200-300-400-500-750	0.05-0.1-0.5-0.75-1-2.5-5-10

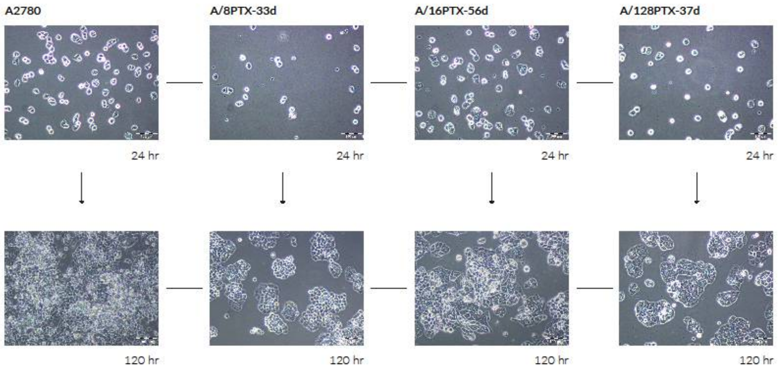

Figure S1. A2780, A/8PTX-33d, A/16PTX-26d and A/128PTX-37d established cell sublines in 24 h and 120 h after seeding.

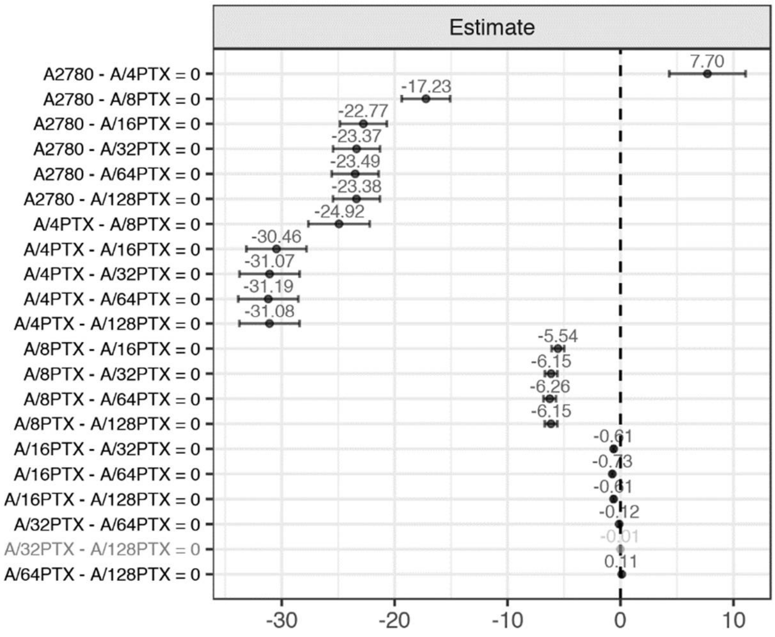

Figure S2. Pairwise comparison of cell lines survival in PTX cytotoxicity assay using 95% CI of the differences between slope parameters (estimates) in LMM. Pairs of cell lines, which differ in statistically significant manner, are indicated in black. Pairs of cell lines, which do not differ in statistically significant manner, are indicated in grey.

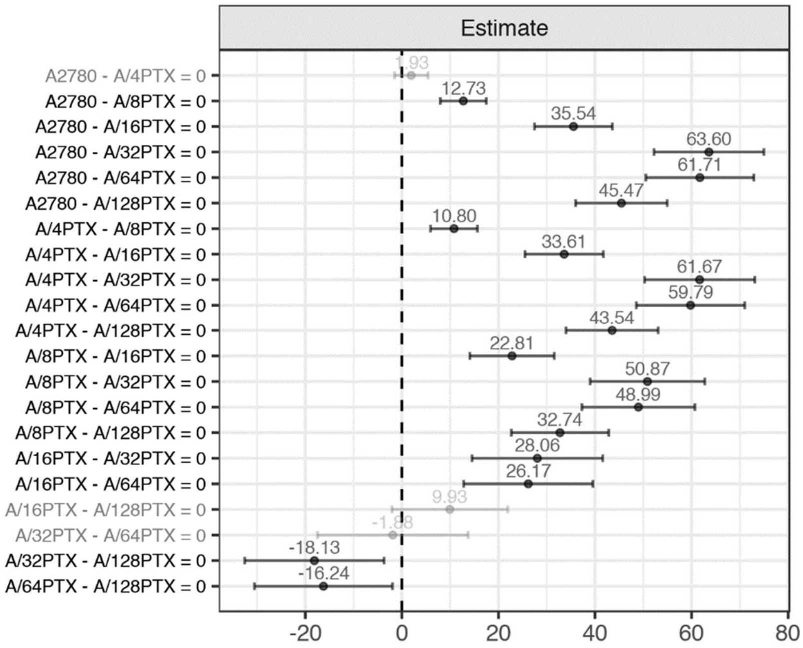

Figure S3. Pairwise comparison of cell lines survival in CDDP cytotoxicity assay using 95% CI of the differences between slope parameters (estimates) in LMM. Pairs of cell lines, which differ in statistically significant manner, are indicated in black. Pairs of cell lines, which do not differ in statistically significant manner, are indicated in grey.

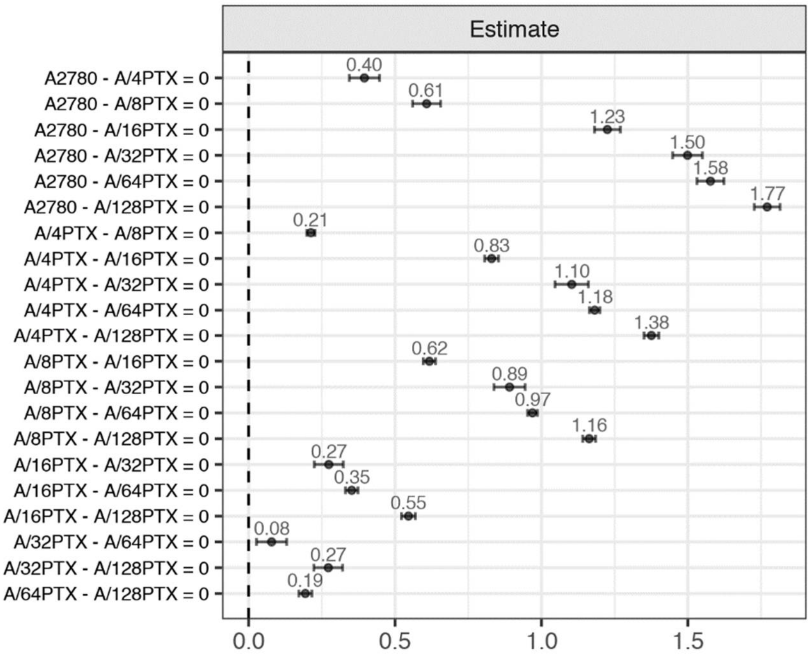

Figure S4. Pairwise comparison of cell lines survival in PTX cytotoxicity assay using 95% CI of the differences between means (estimates) in Student's t-test. All cell line pairs differ in statistically significant manner and they are indicated in black.

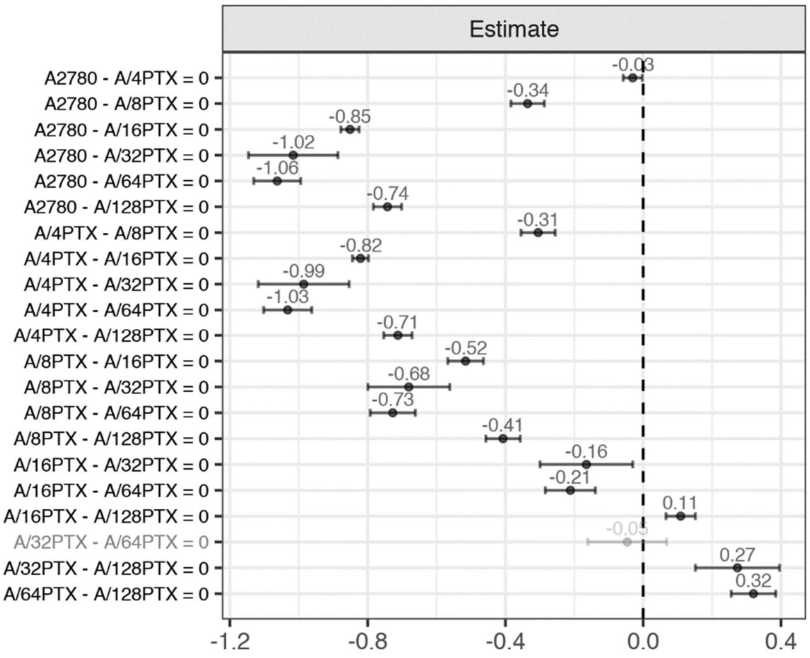

Figure S5. Pairwise comparison of cell lines survival in CDDP cytotoxicity assay using 95% CI of the differences between means (estimates) in Student's t-test. Pairs of cell lines, which differ in statistically significant manner, are indicated in black. Pairs of cell lines, which do not differ in statistically significant manner, are indicated in grey.

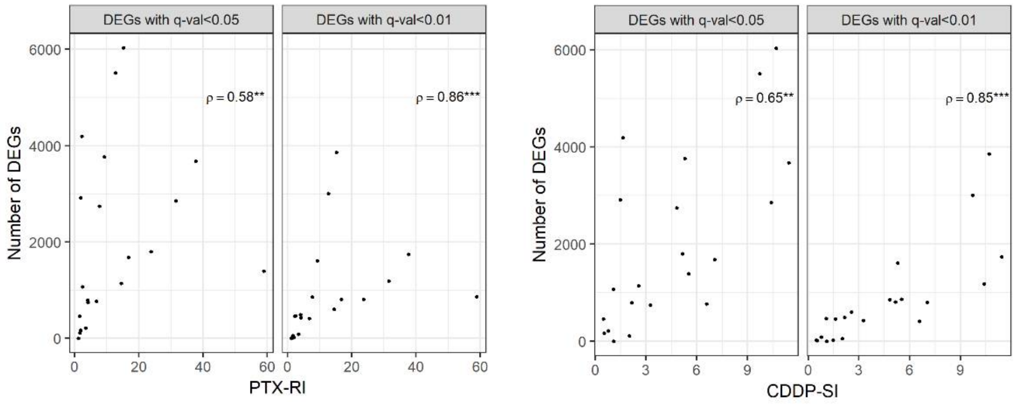

(**A**) (**B**)

Figure S6. The scatterplots illustrating the relationship between fold resistance to PTX (PTX-RI) **(A)** and fold sensitivity to CDDP (CDDP-SI) **(B)** and the number of DEGs retrieved from 21 pairwise comparisons between parental A2780 cell line and six derived sublines. The ϱ denotes Spearman's rank correlation coefficient. Significant codes: ** p-value < 0.01; ***p-value < 0.001.

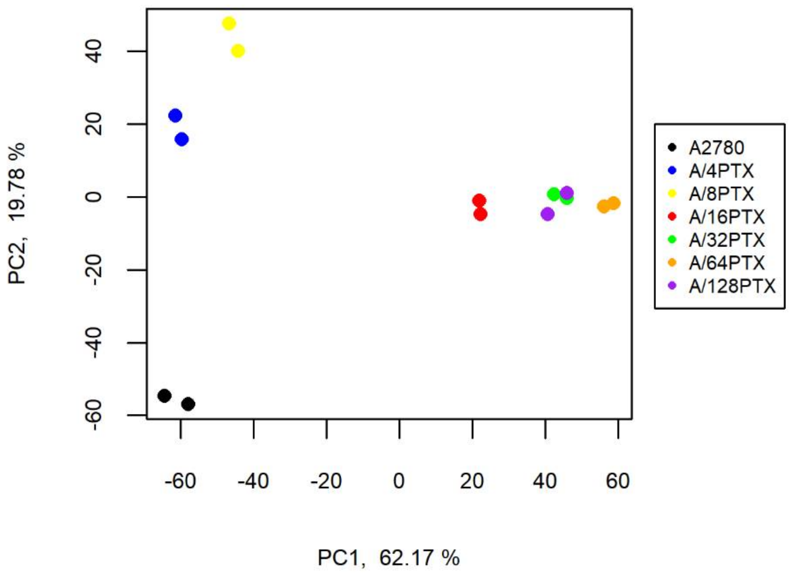

Figure S7. PCA performed on the basis of different gene expression in A2780 cells and its six derivatives with inverse resistance to PTX and CDDP. Analysis was performed on 997 the most significant DEGs (q-value < 0.001), independently on two biological replications for each cell line. A2780 parental cell line and two sublines at the early stages in inverse resistance development (A/4PTX and A/8PTX) are well separated. Four sublines at the late stages (A/16PTX, A/32PTX, A/64PTX and A/128PTX) form a separated cluster, but A/32PTX and A/128PTX sublines are undistinguishable having only two principal components: PC1 and PC2.

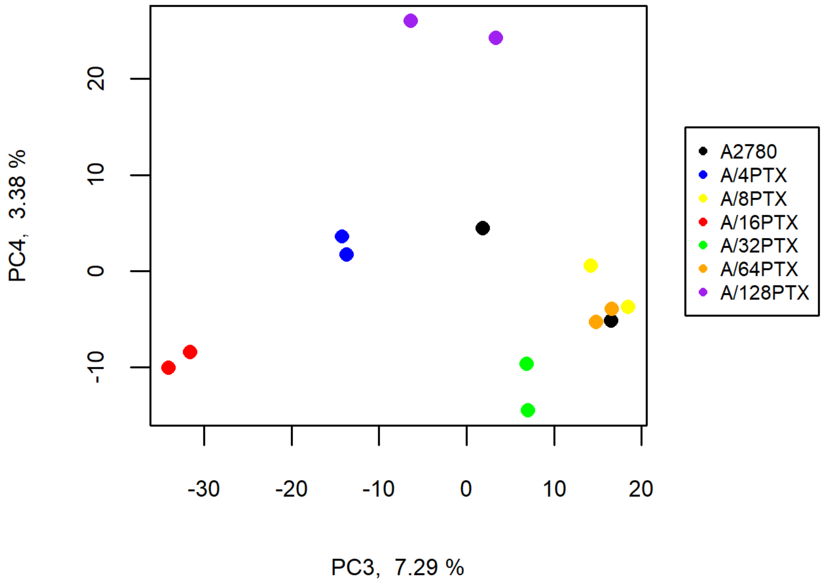

Figure S8. PCA performed on the basis of different gene expression in A2780 cells and its six derivatives with inverse resistance to PTX and CDDP. Analysis was performed on 997 the most significant DEGs (q-value < 0.001), independently on two biological replications for each cell line. Two

sublines: A/32PTX and A/128PTX at the late stages in inverse resistance development, that were not separated with the first two principal components (see Suplementary Figure S7), are distinguishable with PC3 and PC4 components.
